# Assessing the Water Pollution of the Brahmaputra River Using Water Quality Indexes

**DOI:** 10.3390/toxics9110297

**Published:** 2021-11-06

**Authors:** Alina Barbulescu, Lucica Barbes, Cristian Stefan Dumitriu

**Affiliations:** 1 Department of Civil Engineering, Transilvania University of Brașov, 5 Turnului Str., 900152 Brașov, Romania; alina.barbulescu@unitbv.ro; 2 Department of Chemistry and Chemical Engineering, Ovidius University of Constanta, 124 Mamaia Bd., 900527 Constanta, Romania; 3 SC Utilnavorep SA, 55 Aurel Vlaicu Av., 900055 Constanta, Romania

**Keywords:** water pollution, CCME WQI, BC WQI, weighted index, classification, trend

## Abstract

Water quality is continuously affected by anthropogenic and environmental conditions. A significant issue of the Indian rivers is the massive water pollution, leading to the spreading of different diseases due to its daily use. Therefore, this study investigates three aspects. The first one is testing the hypothesis of the existence of a monotonic trend of the series of eight water parameters of the Brahmaputra River recorded for 17 years at ten hydrological stations. When this hypothesis was rejected, a loess trend was fitted. The second aspect is to assess the water quality using three indicators (WQI)–CCME WQI, British Colombia, and a weighted index. The third aspect is to group the years and the stations in clusters used to determine the regional (spatial) and temporal trend of the WQI series, utilizing a new algorithm. A statistical analysis does not reject the hypothesis of a monotonic trend presence for the spatially distributed data but not for the temporal ones. Hierarchical clustering based on the computed WQIs detected two clusters for the spatially distributed data and two for the temporal-distributed data. The procedure proposed for determining the WQI temporal and regional evolution provided good results in terms of mean absolute error, root mean squared error (RMSE), and mean absolute percentage error (MAPE).

## 1. Introduction

Water is an essential resource for human life, but access to fresh water is limited and problematic in many zones worldwide. People should use only clean water [1,2,3]. Still, there are many geographical areas where diseases are directly caused by low water quality [4]. For many decades, governments of different countries failed to ensure the population’s access to drinking water with good qualities and for agricultural use [5,6,7,8]. The urbanization, industrial development, extensive use of chemical fertilizers, defective waste deposition, and collection significantly affect the environmental equilibrium. Water quality refers not only to human needs but also to the ecosystems’ existence. High quantities of organic pollutants enrich the surface waters, producing their deterioration and the loss of the natural equilibrium of the aquatic ecosystems [9,10,11,12,13,14,15,16]. Therefore, monitoring waters’ quality is a must. It involves the study of the physical, chemical, and microbiological parameters [17,18,19].

Since the aquatic systems are exposed to pollution from multiple sources, understanding the spatial and temporal variations in their parameters (physical, chemical, and microbiological) is essential for limiting the input of dangerous substances and mitigating the damages’ effects [20]. Different statistical approaches are known for studying water parameters, involving multivariate techniques that detect the factors that significantly influence water quality, spatio-temporal variations, data reduction, data sampling, and grouping [21,22,23,24,25]. Artificial Intelligence, fuzzy models, Monte Carlo simulation [26,27,28], and arithmetic indexing methods have been utilized to model the water parameters’ ensemble [29,30,31,32].

Water quality indexes (WQIs) are tools designed for describing water quality using chemical, physical, and biological parameters by aggregating the information into a single number [32]. They can quickly and logically express the information on the water quality and help to understand the overall water status at different monitoring sites for various uses [17]. They permit classifying the water into different classes, such as ‘good’, ‘bad’, ‘fair’, ‘poor’, ‘borderline’, etc., [33].

Since 1960, when Horton [34] introduced the first WQI, many other indexes have been proposed for water quality estimation. Until 2019, eleven fundamental models and two groups of modified versions of WQIs were developed [35]. The first group includes six models, of which the Bascaron [36] index and CCME [37] indexes belong. The second one contains three indexes: Dinius [38], Oregon [39], and West Java [40].

Gupta et al. [41] showed that the water indexing systems require measurements realized by selecting water quality parameters. Different values of evaluated sub-indices corresponding to each parameter result from the analysis of the performed measurements. Then, the results are aggregated to obtain the final score of the index corresponding to the quality of the evaluated water.

The parameters that contribute to the WQIs computation are chosen based on several risk aspects identified in the water quality analysis:

- The intensification of the eutrophication process;
- The availability of dissolved oxygen;
- The health assessment of the ecosystems;
- The specific physical and chemical processes occurring in the evaluated water bodies.

Usually, WQIs do not take into account radioactive or toxic elements to assess water quality. However, some methods employed to calculate WQI indices, such as Oregon [39], West Java [40], Almeida [42], Dojildo [43], and Liou [44] recommend the inclusion of the toxic compounds (detergents, phenols, pesticides or metal species, As, Pb, Cd, Hg, Cu, Zn, Fe, Mn, etc.) in the water quality evaluation.

Some of the most used WQIs are the British Colombia WQI (BC WQI) and the Canadian Council Water Quality Index (CCME WQI).

The British Colombia Ministry of Environment, Lands and Parks proposed the British Colombia Water Quality (BCWQI) [45] as the national index and the basis of other provincial indexes. The index was introduced to reduce the amount of information necessary to be communicated to the public, which was difficult to be understood. It was not initially intended to be used by professionals.

CCME was established in 2001, and, since then, it has been utilized in Canada and worldwide to report the water quality throughout the world for evaluating the state of water quality. The CCME WQI is based on the index developed by the British Columbia Ministry of Environment, Lands, and Parks [46] and incorporates modifications created by the province of Alberta, and closely resembles the Alberta Agricultural Water Quality Index [47].

Recently, machine learning algorithms were employed by Granata et al. [48] to evaluate the trend of the wastewater quality indicators based on some characteristics of a drainage basin. Oladipo et al. [49] combined the fuzzy logic (FL) and the WQI to assess the water quality in a zone of Nigeria. Sutadian et al. [40] introduced a new index for the West Java Province. Shah et al. [50] also used artificial intelligence methods to model monthly total dissolved solids and specific conductivity in the upper Indus River.

Other authors [18,51,52] investigated groundwater vulnerability by DRASTIC and multivariate methods. Statistical approaches were utilized by Mamun et al. [53] and Al-Taani et al. [54] in their studies concerning an artificial dam reservoir and the waters of Aqaba Gulf. Chemometrics methods were employed by Yu et al. [55], while the effects of water pollution on human health were investigated by other authors [56,57].

Indian rivers are facing massive pollution [58,59,60,61,62,63]. Ganga, Krishna, Cauvery, Sabarmati are on the top four rivers for wastewater generation. The Brahmaputra, one of the largest Indian rivers, with an average annual runoff of 591 km^3^/year, produces about 179 million liters of wastewater daily [63].

Scientists studied the water quality of these Indian rivers, triggering an alarm signal on the impact of the pollution on the environment and human health. Gupta et al. [41] computed five water quality indexes for assessing the water quality in a port from Bombay, Bora, and Goswami [64] performed the analysis of the Kolong River water quality at various seasonal stages. Bărbulescu et al. [65] and Bărbulescu and Dani [66] investigated the water parameters of the Sutlej and Beas Rivers. Bhargava [62] proposed the zonation of Ganga based on water quality indexes. Chakrabarty and Sarma [67] studied the drinking water contamination in the Asam region, while other scientists [60,68,69,70] performed similar analyses for other major rivers in India. A critical analysis of all these studies reflects the acute need for more careful monitoring and control of the Indian rivers’ water quality [71].

In the above general context, the goal of the present study is to assess the water quality evolution at spatial and temporal scales based on the series of eight water parameters measured at ten hydrological stations on the Brahmaputra River for 17 years. First, the existence of a trend (in time and space) of the water parameters series is investigated. Then, three water quality indicators (WQIs) are computed and used for classifying the water quality at the studied sites (spatial scale) between 2003 and 2019 (temporal scale). The third step is grouping the locations (and years) using the WQIs previously computed utilizing hierarchical clustering. The clusters with the highest number of elements are the input of a new algorithm for determining the water quality trend in time and along the river.

This approach is new for the following reasons: (1) No study has used different water quality indicators for grouping the series in clusters as temporal and spatial scales. Generally, only one water parameter recorded at various locations is analyzed and used for clustering the sites. Here, all the water parameters intervene in computing the WQIs, which, at their turn, are employed for classification. Studies generally report only the temporal or spatial evolution of different water parameters, modeling their trends through various methods. (2) The classification is performed for sites and years, based on the WQIs, not on the series of individual water parameters. So, both temporal and spatial dimensions are considered, and the information provided by the individual water parameters is aggregated in the WQIs. (3) The temporal and regional evolutions of WQIs are determined based on an original algorithm.

## 2. Material and Methods

### 2.1. Study Area and Data Series

**Figure 1:**
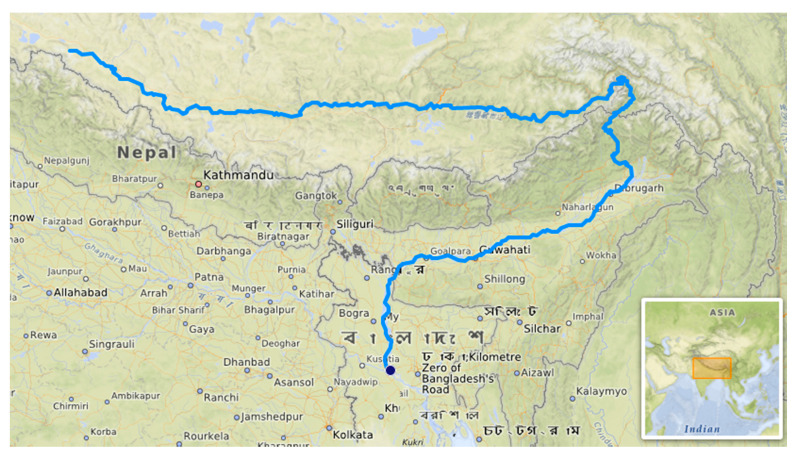
Brahmaputra River map (https://en.wikipedia.org/wiki/Brahmaputra_River#/media/File:Brahmapoutre.png (accessed on 15 September 2021).

The Brahmaputra River (Figure 1), located in South Asia, situated between 23°N and 32°N latitude and 82°E and 97°E longitude is considered the fifth largest river system globally in terms of annual average discharges (about 20,000 m^3^/s) [72].

The basin has a maximum east-west length of 1540 km and north-south width of 682 km. On Indian territory, the Brahmaputra valley is narrow and long, has 640 km length and 64–90 km width [73]. The Brahmaputra basin covers 580,000 km^2^ in India, China, Bangladesh, and Bhutan [74]. It flows into the Bay of Bengal after joining with Ganga. The main canal of the Brahmaputra River crosses China, India, and Bangladesh and is2880 km in length. Three zones of the river basin can be distinguished: the Tibetan Plateau (TP) (with an elevation between 3000 and 5000 m), the Himalayan Belt (with elevations between 100 m and 3500 m), and the floodplain [72].

In the basin area, there are four seasons: the relatively dry-cool, dry-hot, the southwest monsoon, and retreating monsoon during December–February, March–May, June–September, and October–November, respectively.

Climatic conditions influence the annual regime of river flow. On the Indian Territory, the River has 11 main tributaries [75] that experience two high-water seasons, so the agricultural sector suffers from frequent flooding.

The flood’s consequence is a large-scale persistent erosion of the river’s banks. During the rainy season, it causes the breakage of the banks that are not robust enough to cope with the high pressure of the overflowing waters. Silt and sandy materials are carried by waters, affecting the cultivated agricultural lands that become unsuitable for immediate use. Generally, flooding happens during the monsoon season. Several floods have devastated the lands situated in the Brahmaputra basin in the last decades [76].

The yearly sediment load is about 735,000,000 metric tons, while its specific flood discharge is 0.15 m^3^/s/km^2^.

The soils in the Brahmaputra basin are Lithosols (in the Tibetan Plateau), orthic acrisols (in the Himalayan belt), and eutric cambisols and eutric gleysols (in the floodplain) [76].

The potentially usable water resources are estimated at 50 km^3^/year, out of which about 90% remain unutilized. The river’s waters are mainly utilized for irrigation (81%), household use (10%), and in the food (9%) industry [77].

Data series were downloaded from the site of ENVIS Centre on Control of Pollution Water, Air, and Noise [78]. They are data from official reports of the Ministry of Environment and Forests from India and contain the annual series of temperature (°C), pH, BOD (mg/L), DO (mg/L), electrical conductivity (EC) (µmhos/cm), Nitrate and Nitrite (mg/L), (fecal coliform (FC) (MPM/100 mL), and total coliform (TC) (MPM/100 mL) collected from 2003 to 2019 at ten hydrological stations (denoted in the following by S1–S10) situated on the Brahmaputra River.

### 2.2. Preliminary Statistical Analyses

The boxplots for the water parameters were drawn to detect the series variability and the outliers’ existence.

To verify the hypothesis that there is no trend against the existence of a monotonic trend of a particular series of water parameters, the Mann–Kendall trend test [79] was used, followed by the nonparametric procedure of Sen [80] if the null hypothesis was been rejected.

The Kruskal–Wallis test was performed to determine if the series of a specific pollutant recorded at different sites come from the same distribution [81].

A loess trend was built to emphasize the evolution of each series of water parameters over the entire study period. In this procedure, for fitting the values at a point, the values from its neighbors are utilized weighted by the distance between the target point and the neighbor. A parameter α controls the size of the neighborhood. For α < 1, the neighborhood includes a proportion α of the points, with tricubic weighting [82]. In this analysis, α was chosen 0.10, 0.25, and 0.50, for comparison reasons.

### 2.3. The Water Pollution Indices

Three water quality indexes were computed to evaluate the water pollution at each station and the yearly pollution along the river. They are the Canadian Council Water Quality Index (CCME WQI) [83], the British Columbia Water Quality Index (BC WQI) [84], and the arithmetic weighted index [39]. These indexes are defined in the following.

CCME WQI is computed by:

(1) $$\mathrm{CCME}\;\mathrm{WQI}=100-\sqrt{F_{1}^{2}+F_{2}^{2}+F_{3}^{2}}/1.732$$

where

(a) *F*_1_ is the ratio between the number of the failed parameters and the total number of parameters, multiplied by 100;
(b) *F*_2_ is the ratio between the number of the failed tests and the total number of tests, multiplied by 100.

(2) $$F_{3}=nse/\left(0.01nse+0.01\right)$$

where *nse* is obtained by dividing the sum of individual excursions by the total number of tests.

An individual excursion is computed by:

(3) $$excursion_{i}=\frac{Failed\;test\;value_{i}}{Objective_{j}}-1$$

If a test value falls below the objective value, and:

(4) $$excursion_{i}=\frac{Objective_{j}}{Failed\;test\;value_{i}}-1,$$

If a test value exceeds the objective value.

BC WQI is defined by [81]:

(5) $$\mathrm{BCWQI}=\sqrt{F_{1}^{2}+F_{2}^{2}+\left(\frac{F_{3}^{2}}{9}\right)}/1.453$$

where

*F*_1_ = number of objectives not met/total number of objectives × 100, *F*_2_ = frequency of objectives not met/all instances of the objectives × 100, *F*_3_ = maximum deviation from any objectives.

Based on the CCME WQI, the following classes of water quality are determined: 95–100, Excellent; 80–94, Good; 65–79, Fair; 45–64, Marginal; and 0–44, Poor.

Based on the CCME WQI, the water categories are 0–3, Excellent; 4–17, Good; 18–43, Fair; 44–59, Borderline; 60–100, Poor.

The arithmetic weighted index [39] is defined by:

(6) $$WWQI=(\sum_{i=1}^{n}w_{i}Q_{i}\;)/\left(\sum_{i=1}^{n}w_{i}\right),$$

where *w_i_* is the weight corresponding to the quality index associated to the *i*th parameter,

(7) $$Q_{i}=100\times \left(V_{i}-V_{0}\right)/\left(S_{i}-V_{0}\right)$$

*V*_0_ =7.0 for the pH, *V*_0_ = 14.6 mg/L for DO, and *V*_0_ = 0 for the other water parameters, *V_i_* is the concentration of *i*th water parameter, *S_i_* is the standard value of the *i*th parameter and:

(8) $$w_{i}=\frac{1/S_{i}}{\sum_{i=1}^{n}\left(1/S_{i}\right)}$$

The water quality is Excellent, Good, Poor, Very Poor, or Unsuitable for drinking if the weighted arithmetic index is in the ranges (0–25), (26–50), (51–75), (76–100), and (above 100), respectively.

### 2.4. Classification

The sets of the WQIs computed at the previous stage were utilized to group the stations (respectively, the yearly series) in different clusters using agglomerative hierarchical clustering [85]. The optimal number of clusters was determined based on the majority principle after running 28 selection algorithms implemented in the NbClust package in R software [86].

### 2.5. Determination of the WQI Trend in Time and over the Region

The following procedure was utilized to determine the regional trend of the WQIs. This is a version of Method II from [25], where the k-mean clustering is replaced by the hierarchical clustering.

Suppose that *k* data series registered in *n* consecutive periods are provided and let us denote by (*y_ji_*) (*j =* 1, …, *n*) the series registered at the station *i* (*i =* 1, …, *m*).

(II1) Choose the number of clusters and perform the clustering;

(II2) Determine the cluster containing the highest number of elements and build a matrix using the data series recorded at the sites from that cluster;

(II3) Choose the value representing the row *j* to be the average of the values recorded at the moment *j* at the stations from the cluster with the highest number of observations;

(II4) Represent graphically the results;

(II5) Compute the mean absolute error (MAE) and Mean Standard Error (MSE) and mean absolute percentage error (MAPE) corresponding to all the observation sites to assess the goodness-of-fit of the regional series.

The same procedure is applied for assessing the temporal trend of the WQI. In this case, the involved matrix (*y_ji_*)’ is the transposed of (*y_ji_*) from the above algorithm, so the sites are replaced by the periods and vice-versa.

In both cases, the procedure is applied for the WQIs yearly computed by the weighted index.

## 3. Results and Discussion

### 3.1. Statistical Analysis

**Figure 2:**
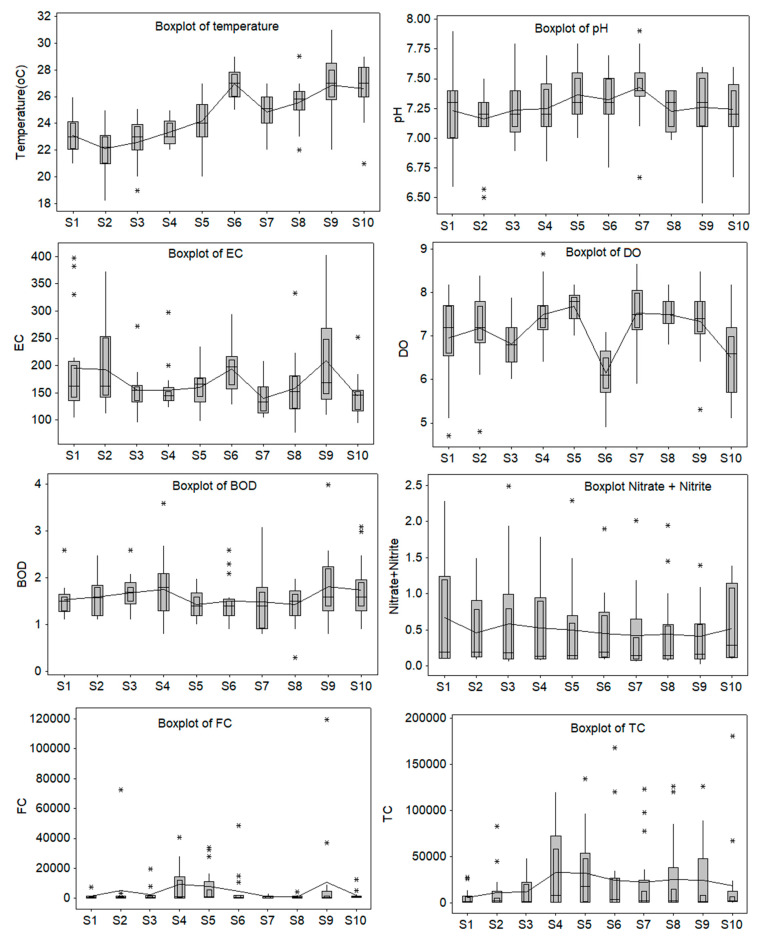
Boxplots of the water parameters; from top to bottom and left to right: temperature, pH, EC, DO, BOD, Nitrate and Nitrite, FC, and TC.

Figure 2 displays the boxplots of the study parameters recorded at the stations S1-S10. All series present outliers. Notice the high values recorded for TC and FC at S5-S10, and FC at S9, S2, S6, and S5. Some extreme values of EC are present at S1 and for BOD at S9, S10, and S10. Thus, these values negatively impact the WQI.

After performing the Mann–Kendall test, the null hypothesis was rejected for most series. Table 1 contains the results of Sen’s slope estimation for the water parameters series registered at the hydrological stations. The positive values indicate an increasing trend; the negative ones point out a decreasing trend, whereas ‘-’ means that the null hypothesis cannot be rejected.

Table 1 shows that the series of nitrate and nitrites have an increasing trend at all the stations, while the EC trend is decreasing at six out of ten sites. The TC series does not present a trend. FC has an accentuated negative slope at S1 and a small one at S8. Overall, the highest variability of the water parameters is noticed at S1, followed by S8.

The Kruskal–Wallis test applied to the series of the same parameter collected at different stations rejected the null hypothesis only for temperature, EC, and DO.

Only a few series present a trend: temperature in 2008, 2009, 2011–2014, 2016–2019, EC in 2006 and 2010, and FC in 2018. There is only one series with a negative trend, EC (in 2006). So, the spatial variability is more accentuated than the temporal one. Taking into account these results, one might expect slight variations in the values of water quality indicators.

The Kruskal–Wallis test applied to the annual series of parameters rejected the null hypothesis for all water parameters but temperature and DO. This means that significant differences among the annual evolution of the water parameters were found.

Table 2 presents the slope evaluation for the yearly series for which the null hypothesis of the Mann–Kendall test was rejected.

**Figure 3:**
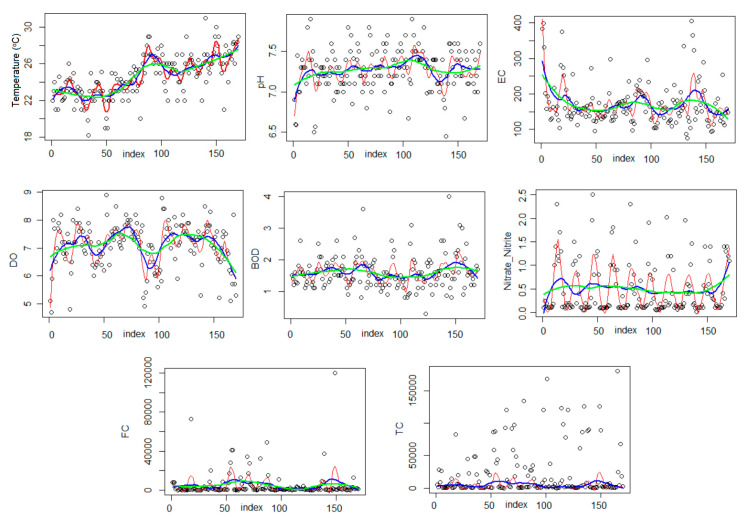
Loess trend for spatio-temporal variation in the water parameters. The red curve corresponds to α = 0.10, the blue one, to α = 0.25, and the green one, to α = 0.50.

To have a complete image of the spatial and temporal variation in the water parameters, the loess curve was fitted for each series, with different values of the parameter α. The blue curve in Figure 3 corresponds to α = 0.10, the blue one to α = 0.25, and the green one to α = 0.50.

The loess curves for α = 0.10, (red) presents a periodical behavior for almost all series, with the highest variation for DO and Nitrate and Nitrite. Compared with the other loess curves (green and blue), their amplitudes are higher. This means that the influence of pollution in the locations closer to the analyzed site is more significant than the influence of the concentrations recorded at longer distances.

Figure 3 shows that the pollution is not uniformly distributed along the river, and overall, the pollution did not decrease during the study period.

### 3.2. WQIs Computation

**Figure 4:**
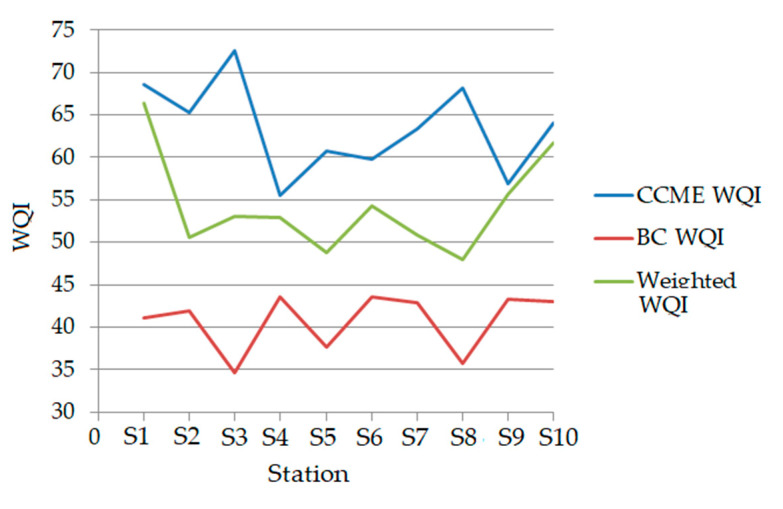
Water quality indexes computed for each station.

The values of the water quality indicators computed at the hydrological stations are presented in Figure 4. Table 3 contains the waters’ classification based on the calculated indexes.

Based on the CCME WQI, all but the water samples are classified as marginal or fair. Based on the BC WQI, they are fair or fair to borderline, whereas the water quality falls in the categories, poor or good, based on the weighted index.

**Figure 5:**
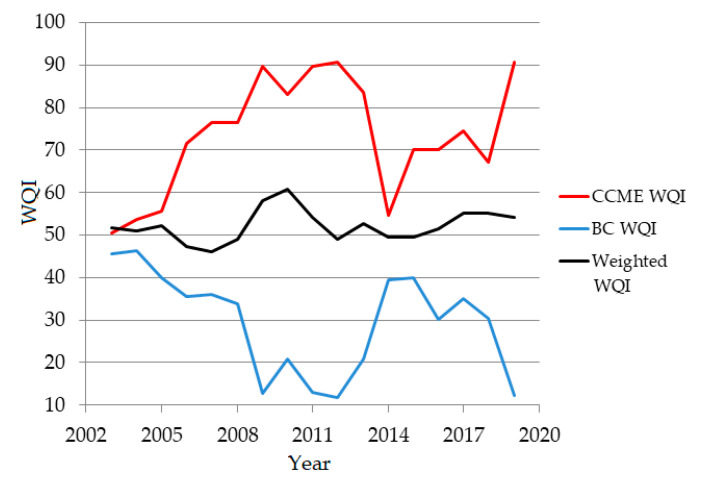
Water quality indexes computed for yearly series.

The WQIs for the temporal series are represented in Figure 5. Table 4 contains the WQIs values for the annual series. No improvement in the water quality was noticed over the years. Moreover, a decrease in its quality appeared after 2015.

### 3.3. Clustering Data Series

**Figure 6:**
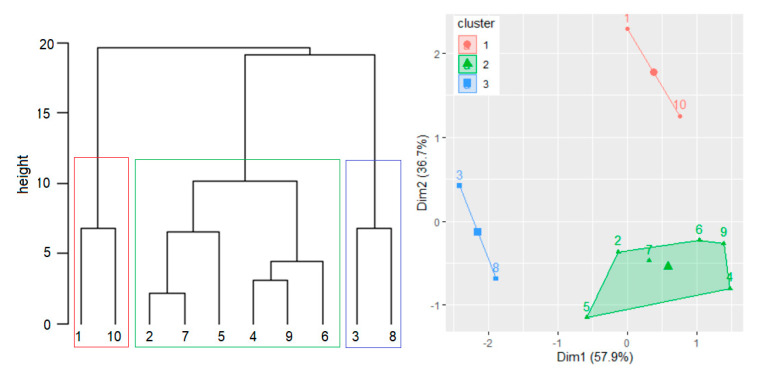
Dendrogram and clusters built using the WQIs from S1–S10.

In the hierarchical clustering performed after scaling the WQIs from Table 3, three clusters were utilized. This number was determined by running 28 algorithms, among which ten selected three as the optimal value of the numbers of groups. The corresponding agglomerative coefficient was 0.755. Figure 6 displays the dendrogram and the clusters obtained.

The first and third clusters contain only two stations, whereas the second one has six elements. The four variables utilized to build CCME WQI do not meet the objectives for S1 and S10.

The null hypothesis could not be rejected by the Kruskal–Wallis test for the average value of the eight water parameters registered at S1 and S10. The same is true for the series in the other two clusters, confirming the correct clustering.

Hierarchical clustering was performed (after scaling the computed WQIs) using two clusters because 10 out of the 28 methods performed for finding the optimal number of groups found this number (two groups).

For measuring the clustering amount, the agglomerative coefficient was computed as well. Since its value was 0.947, the clustering is good.

**Figure 7:**
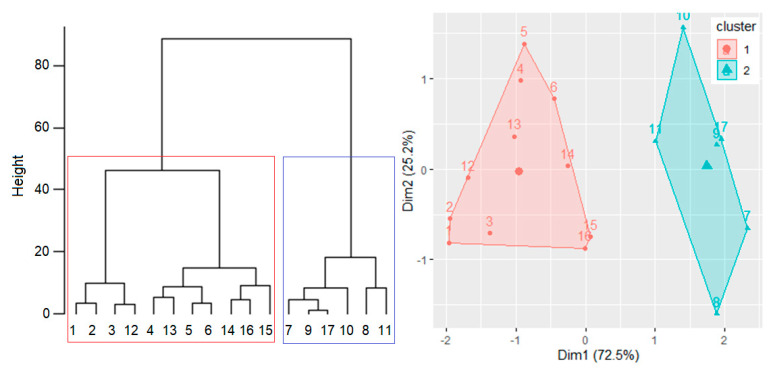
Dendrogram and clusters built using the yearly WQIs.

The dendrogram produced by the agglomerative algorithm and the clusters are presented in Figure 7.

The series contained in the second cluster are characterized by values of FC and TC under the admissible limits, CCME WQI good, and BC WQI, good or fair.

### 3.4. Determination of the Regional Series and Temporal ‘Global’ Series

**Figure 8:**
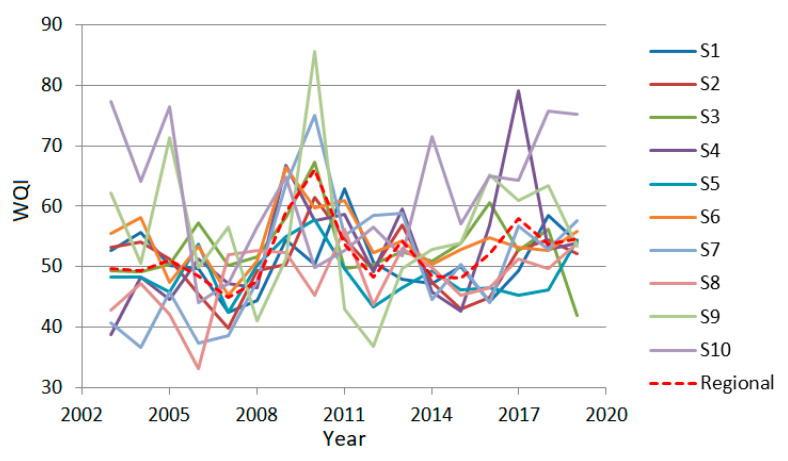
The regional WQI series.

The ‘regional’ series is the series that describes the WQI trend at the spatial scale. It is represented in Figure 8 by the red line and was computed as described in the first part of Section 2.5. The goodness of fit indicators are provided in Table 5.

All the MAE, RMSE, and MAPE values are small, showing a good fitting of the regional trend of WQIs. The MAPE values are the smallest. Since MAPE is not a dimensional indicator, it is most suitable for assessing the modeling quality.

**Figure 9:**
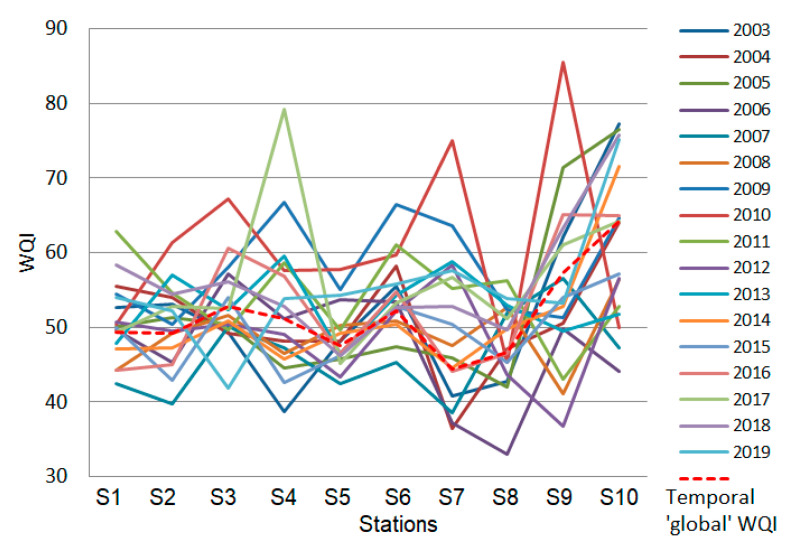
The temporal ‘global’ WQI series.

The temporal ’global’ series is the series that describes the WQI evolution in time, computed as presented in the second part of Section 2.5. It is represented in Figure 9 by the red line. The goodness of fit indicators are provided in Table 6.

In Table 6, MAEs are under 12.60, RMSE under 15.77, and MAPE under 2.14, indicating a good fit of the temporal ‘global’ series. The lowest fit quality is noticed for 2011, in terms of MAPE, and 2010, in terms of MAE and MSE. These are due to a better quality (higher QWI) recorded at the stations S1 and S9, respectively.

From Figure 8 and Figure 9, one can conclude that the water quality varies between good and poor. This is mainly due to the high concentrations of the fecal and total coliforms accumulated in time along with specific sectors of the river.

### 3.5. Discussions of the Present Results Compared with Previous Research

The above results showed that the quality of the Brahmaputra River is not very good. There is a concordance of the water quality classification based on all the used indexes. The results would be more precise if other water parameters were available and taken into account.

Still, our findings do not differ from those of other researchers. For example, Muyen et al. [87] analyzed the pollution of the Brahmaputra River in a sector from Bangladesh in April 2015, and found that the water is highly polluted. Kotoky and Sharma [88] confirmed this idea in a study performed in India in March 2017. They included the water to be in class IV (based on the used WQI). Mech and Hazarika [89] emphasized the impact of industrial effluents on the ecosystem and population lives near Brahmaputra Cracker. Tsering et al. [90] showed that the level of pollution of Brahmaputra with microplastics was extremely high in 2018–2019. The official report on the water quality scenario of rivers [91] emphasizes the increase in water turbidity during 2006–2019. The United Nations, through the Environment Program [92], drew a signal of alarm on the accelerated consequences of the Brahmaputra River’s pollution.

## 4. Conclusions

WQIs are mainly used to assess water quality over a long period and as a tool for making informed decisions on water management policy in water scarcity conditions. Even if there is no mathematical formula to estimate the risk of water consumption based on WQIs, a high WQI class means low risk for the population that consumes the water. For example, when working with the weighted index, if the water is classified as excellent or good, there is no risk for population’s health by its consumption.

Regulations establish allowable limits of water parameters. Since different water parameters can sometimes have values outside the permissible limits, these values should be observed. For example, high values of coliforms may result in diseases after water consumption. Therefore, the WQI use should be correlated with observation of the water parameters.

Other indicators are utilized to assess water use suitability for other purposes, such as agriculture. The series of Na, Cl, bicarbonate ions, Mg, Mn phosphates, and TDS concentrations are necessary to compute such indicators. Unfortunately, these data are not available on the official site [78] from where the other series were downloaded, which would permit us to perform the study in this direction. Still, an integrated analysis of water parameters and WQIs is the best approach for deciding water use for different activities.

This research investigated the series of eight water parameters recorded for 17 years to assess the water quality at the spatial and temporal scales. Based on the CCME WQI, the water quality was Fair (at S1, S2, S3, and S8) and Marginal (the other stations). Based on the BC WQI, the water was classified as Fair or Fair/Borderline. Based on the weighted index, the water was classified as either Poor or Good. The values of the WQIs computed for the annual series indicate a water quality decrease after 2015.

Two clusters were detected based on the computed WQIs for the annual series and three groups for the WQI series corresponding to the hydrological stations, employed to evaluate the WQI trend at the temporal and spatial scales. The water quality is mainly affected by the high concentrations of coliform that exceed many times the legal limits at some stations during the period 2003–2019.

This approach combined the statistical analysis, the computation of the water quality indicators, classification, and trend modeling to evaluate the water quality of the Brahmaputra River. We intend to extend the research by involving more water indicators and other techniques to assess water quality better.

## Figures and Tables

**Table 1:** Sen’s slope evaluation for the series of water parameters registered at the hydrological series.

Sen Slope	S1	S2	S3	S4	S5	S6	S7	S8	S9	S10
Temperature	0.150	−0.221	-	-	-	−0.100	-	-	0.293	0.200
pH	0.047	-	-	−0.027	-	-	-	-	-	-
EC	−10.500	−9.076	-	-	−3.632	−4.903	-	−4.819	−12.667	-
DO	-	-	-	-	-	-	−0.100	−0.044	-	-
BOD	-	-	-	0.071	-	-	-	-	-	-
Nitrate and Nitrite	0.084	0.028	0.055	0.052	0.041	0.020	0.021	0.044	0.049	0.046
FC	−144.792	-	-	-	-	-	-	−0.833	-	-
TC	-	-	-	-	-	-	-	-	-	-

**Table 2:** Sen’s slope evaluation for the yearly series of water parameters.

Year	2003	2004	2005	2006	2007	2008	2009	2010	2011
Temperature	-	-	-	-	-	0.714	0.275	-	0.456
pH	-	-	-	-	-	-	-	-	-
EC	-	-	-	−10.500	-	-	-	4.500	-
DO	-	-	-	-	-	-	-	-	-
BOD	-	-	-	-	-	-	-	-	-
Nitrate and Nitrite	-	-	-	-	-	-	-	-	-
FC	-	-	-	-	-	-	-	-	-
TC	-	-	-	-	-	-	-	-	-
**Year**	**2012**	**2013**	**2014**	**2015**	**2016**	**2017**	**2018**	**2019**	
Temperature	0.600	0.500	0.656	-	0.500	0.833	0.667	1.000	
pH	-	-	-	-	-	-	-	-	
EC	-	-	-	-	-	-	-	-	
DO	-	-	-	-	-	-	-	-	
BOD	-	-	-	-	-	-	-	-	
Nitrate+Nitrite	-	-	-	-	-	-	-	-	
FC	-	-	-	-	-	-	172.000	-	
TC	-	-	-	-	-	-	-	-	

**Table 3:** Water quality indexes at each station for the study period.

Station	CCME WQI		BC WQI		Weighted WQI	
	Value	Class	Value	Class	Value	Class
S1	68.56	Fair	41.07	Fair	66.42	Poor
S2	65.33	Fair	41.93	Fair	50.61	Good/Poor
S3	72.54	Fair	34.68	Fair	53.03	Poor
S4	55.59	Marginal	43.57	Fair/Borderline	52.89	Poor
S5	60.81	Marginal	37.63	Fair	48.74	Good
S6	59.74	Marginal	43.53	Fair/Borderline	54.35	Poor
S7	63.43	Marginal	42.92	Fair	50.81	Good/Poor
S8	68.12	Fair	35.71	Fair	48.03	Good
S9	56.89	Marginal	43.31	Fair/Borderline	55.72	Poor
S10	64.02	Marginal	43.07	Fair/Borderline	61.78	Poor

**Table 4:** Water quality indexes for annual series.

Station	CCME WQI		BC WQI		Weighted	
	Value	Class	Value	Class	Value	Class
2003	50.60	Marginal	45.46	Bordeline	51.66	Poor
2004	53.74	Marginal	46.21	Bordeline	50.85	Good/Poor
2005	55.67	Marginal	40.03	Fair	52.23	Poor
2006	71.58	Fair	35.43	Fair	47.34	Good
2007	76.37	Fair	36.00	Fair	46.15	Good
2008	76.37	Fair	33.77	Fair	48.91	Good
2009	89.74	Good	12.84	Good	58.19	Poor
2010	83.13	Good	20.87	Fair	60.84	Poor
2011	89.74	Good	12.98	Good	54.25	Poor
2012	90.73	Good	11.81	Good	48.91	Good
2013	83.49	Good	20.76	Fair	52.72	Poor
2014	54.67	Marginal	39.43	Fair	49.53	Poor
2015	70.09	Fair	39.96	Fair	49.46	Good
2016	70.09	Fair	30.25	Fair	51.54	Poor
2017	74.40	Fair	34.99	Fair	55.09	Poor
2018	67.06	Fair	30.27	Fair	55.06	Poor
2019	90.70	Good	12.33	Good	54.13	Poor

**Table 5:** Goodness of fit indicators for the regional series modeling.

	S1	S2	S3	S4	S5	S6	S7	S8	S9	S10
MAE	4.72	3.42	3.92	5.16	4.47	4.05	5.51	6.05	8.50	12.44
RMSE	6.10	4.02	5.04	6.96	5.34	4.64	6.49	7.66	9.94	14.61
MAPE	0.33	0.38	0.05	1.67	0.19	0.61	1.30	0.95	1.18	2.10

**Table 6:** Goodness of fit indicators in the temporal ’global’ WQI series modeling.

	2003	2004	2005	2006	2007	2008	2009	2010	2011	
MAE	5.25	3.94	5.06	6.45	6.39	4.80	8.02	12.60	8.67	
RMSE	6.52	4.78	6.69	8.81	7.64	6.55	9.97	15.77	9.51	
MAPE	0.63	1.11	0.15	0.04	1.64	1.15	0.92	0.20	2.14	
	**2012**	**2013**	**2014**	**2015**	**2016**	**2017**	**2018**	**2019**		
MAE	5.56	6.22	3.02	3.64	3.58	5.57	5.00	6.73		
RMSE	8.46	7.77	3.63	4.63	4.56	9.94	6.10	7.62		
MAPE	0.27	0.32	0.47	0.12	1.18	0.00	1.54	0.86		

## Data Availability

http://www.cpcbenvis.nic.in/water_quality_data.html.
